# Case Report: *In Situ* Vaccination by Autologous CD16^+^ Dendritic Cells and Anti-PD-L 1 Antibody Synergized With Radiotherapy To Boost T Cells-Mediated Antitumor Efficacy In A Psoriatic Patient With Cutaneous Squamous Cell Carcinoma

**DOI:** 10.3389/fimmu.2021.752563

**Published:** 2021-12-23

**Authors:** Jun-Wei Huang, Chun-Lin Kuo, Li-Tzu Wang, Kevin Sheng-Kai Ma, Wen-Yen Huang, Feng-Cheng Liu, Kuender D. Yang, Bing-Heng Yang

**Affiliations:** ^1^ Division of Family Medicine, Department of Family and Community Medicine, Tri-Service General Hospital, Taipei, Taiwan; ^2^ Department of General Medicine, Tri-Service General Hospital, Taipei, Taiwan; ^3^ Department of Obstetrics & Gynecology, National Taiwan University Hospital & College of Medicine, Taipei, Taiwan; ^4^ Department of Life Science, National Taiwan University, Taipei, Taiwan; ^5^ Center for Global Health, Perelman School of Medicine, University of Pennsylvania, Philadelphia, PA, United States; ^6^ Department of Radiation Oncology, Tri-Service General Hospital, Taipei, Taiwan; ^7^ Division of Rheumatology/Immunology and Allergy, Department of Medicine, Tri-Service General Hospital, Taipei, Taiwan; ^8^ Department of Microbiology & Immunology, National Defense Medical Center, Taipei, Taiwan; ^9^ Institute of Clinical Medicine, National Yang-Ming University, Taipei, Taiwan; ^10^ Division of Allergy, Immunology & Rheumatology, Mackay Children’s Hospital, Taipei, Taiwan; ^11^ Division of Clinical Pathology, Department of Pathology, Tri-Service General Hospital, Taipei, Taiwan; ^12^ Trace Element Research Center, Department of Pathology, Tri-Service General Hospital, Taipei, Taiwan; ^13^ Graduate Institute of Medical Sciences, National Defense Medical Center, Taipei, Taiwan

**Keywords:** *in situ* vaccination, dendritic cell-based immunotherapy, checkpoint inhibitor combination therapy, radiotherapy, monocyte-derived dendritic cells (MoDCs), intratumoral injection therapy, psoriasis vulgaris (PV), cutaneous squamous cell carcinoma (cSCC)

## Abstract

The combination of radiotherapy and immunotherapy improves the survival rate of patients with malignancies developed through escape from T-cell-mediated immune surveillance. Immune checkpoint inhibitors, such as anti-programmed cell death protein-ligand 1 (anti-PD-L1) antibody, are used to rescue exhausted T cells. Simultaneously, dendritic cells (DCs) which are antigen-presenting cells that can initiate T-cell activation, are used to induce a tumor-specific immune response. However, the synergistic antitumor efficacy of the aforementioned combinational immunotherapy with intratumoral injection of low-dose DCs has not been reported, and the underlying therapeutic mechanism requires further investigation. Herein, we present the special case of a psoriatic patient with cutaneous squamous cell carcinoma (cSCC) in the right inguinal region, these two diseases characterized by opposing contradiction, further complicating treatments and side-effect management efforts. To treat the intractable SCC without exaggerating psoriasis, we developed the triple-regimen therapy (TRT) with the intratumoral injection of low-dose autologous DCs and anti-PD-L1 combined with radiotherapy. The injected DCs were obtained simply through leukapheresis without prior G-CSF administration for mobilization nor tumor-antigen loading for expansion. The patient received three radiation doses (24, 18, and 18 Gy) combined with three intratumoral injections of anti-PD-L1 antibody (40, 60, and 120 mg) plus autologous DCs (80% of the DC subpopulation being CD16^+^ myeloid DC with approximate amounts of 7.3 × 10^4^, 2.5 × 10^6^, and 1.7 × 10^7^) within 10 weeks. The efficacy of the TRT was encouraging in shrinking tumor mass with remarkable SUVmax reduction (approximately 42%) on FDG PET-Scan despite relatively low-dose DCs were available. The low-dose intratumoral immunotherapy induced mild cutaneous side effects as expected. The transcriptomes were compared between pre-TRT and post-TRT biopsies to analyze underlying mechanical pathways of the TRT protocol. Over 10 highly significantly enriched T-cell-related pathways (*P <*0.0001) were identified in post-TRT biopsies. In addition, the activation of both innate and adaptive immunity was significantly enriched in post-TRT peripheral blood samples. We develop the easily accessible TRT which produces both local anti-tumor T-cell responses and systemic antitumor immunity for treating cSCC patients, especially for those with autoimmune disease.

## Introduction

Cutaneous squamous cell carcinoma (cSCC), which accounts for 20% of cases of non-melanoma skin cancer, has a strong metastatic capability and thus a high mortality rate (1). Surgical excision is primarily adopted for localized cSCC, and radiotherapy or chemotherapy is used for locally advanced cSCC with nonresectable tumors or metastatic cSCC (2). Immunotherapy uses immune checkpoint inhibitors (ICIs) such as anti-programmed cell death protein 1 (anti-PD-1) antibody, which is known to enhance antineoplastic immune responses by reducing *T-cell* exhaustion and then restimulating T cells (3). Anti-PD-1 antibody has been tested in a phase 1 trial for advanced cSCC and a phase 2 trial for metastatic cSCC (4) as an alternative management strategy for patients with cSCC and surgical contraindication. Moreover, the efficacy of dendritic cell (DC)-based immunotherapy has been demonstrated in clinical trials for cancers other than cSCC (5). However, the limitations of immunotherapy include low PD-ligand (PD-L) expression levels in some patients and high costs for immune cell processing. In this article, we describe the highly personalized protocol for enriching CD16^+^ myeloid DCs (mDCs) from the peripheral blood (PB) of a patient with PV who developed advanced cSCC. This treatment was implemented through a convenient process and exhibited efficacy for latent-stage cancer, when DC-enriched blood cells were combined with anti-PD-L1 immunotherapy plus radiotherapy.

## Case Description

This is a 63-year-old man with a history of hepatitis B virus (HBV)-related cirrhosis with liver function impairment and also psoriasis vulgaris (PV) for more than 25 years. Notably, there was an unusual feature presented with a palpable mass in the right inguinal region that had reportedly existed for 3 months. Local excision of the subcutaneous mass was performed with poor wound healing during regular follow-up. The patient subsequently developed regional progression, with magnetic resonance imaging revealing a cystic lesion measuring 5.6 cm × 4.2 cm in the axial dimension and 9 cm in craniocaudal distance from the tumor encasement of the right superficial femoral artery. Repeated percutaneous transluminal angioplasty with excision biopsy of the tumor was conducted because of several episodes of massive bleeding, which was possibly caused by pseudoaneurysm rupture. Pathological findings revealed moderately differentiated SCC of soft tissue with 65% expression of PD-L1. Positron emission tomography (PET) was further arranged, which revealed an irregularly shaped fluorodeoxyglucose (FDG)-avid lesion in the right inguinal region (>10 cm) with clinical staging T2N0M0 according to the eighth edition of the American Joint Committee on Cancer Staging Manual.

For this case, complete surgical excision was not possible because the tumor had caused vascular encasement; moreover, systemic chemotherapy is not recommended for the prevention of HBV reactivation (6). After a multidisciplinary discussion, triple-regimen therapy (TRT) comprising ICI, DC, and radiotherapy was initiated with signed consent (2-108-05-119_TSGHIRB).

**Figure 1:**
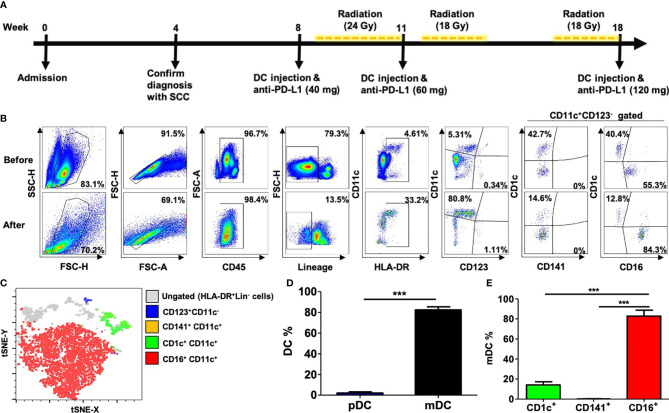
Peripheral blood (PB) CD16^+^ myeloid dendritic cell (mDC) enrichment of a patient with psoriasis and cutaneous squamous cell carcinoma (cSCC). **(A)** Protocol of intratumoral injection of autologous DC-enriched PB plus anti-PD-L1 antibody combined with local radiation. **(B)** Population of mDC and plasmacytoid DC (pDC) in PB before versus after DC enrichment. Blood cells were first gated on FSC-H versus SSC-H, single cells were gated on FSC-A versus FSC-H, and CD45^+^ leukocytes were gated for DC analyses in Lin^−^ (CD3^−^CD14^−^CD19^−^CD20^−^CD56^−^) and HLA-DR^+^ cells. CD11c^+^ and CD123^+^ were then respectively used to identify mDCs and pDCs, with subsequent analyses of CD1c^+^, CD16^+^, and CD141^+^ mDC subpopulations. **(C)** Dimensionality reduction in multiparametric flow cytometry for pDCs, CD1c^+^ mDCs, CD16^+^ mDCs, and CD141^+^ mDCs in Lin^−^HLA-DR^+^ DC-enriched PB, assessed with a t-distributed stochastic neighbor embedding (t-SNE)-based algorithm. **(D)** Pooled data for frequencies of CD123^+^ pDCs and CD11c^+^ mDCs in the Lin^−^HLA-DR^+^ myeloid cells assessed through flow cytometry. ***P <0.001 using an unpaired t test. **(E)** Pooled data for frequencies of CD1c^+^, CD16^+^, or CD141^+^ cells in the Lin^−^HLA-DR^+^CD11c^+^mDC population of DC-enriched PB assessed using flow cytometry. ***P <0.001 using one-way analysis of variance with Tukey’s multiple comparison test.

The therapeutic schedule and regimens are presented in **Figure 1A**. Generally, DC immunotherapy requires multiple steps, namely, leukapheresis, elutriation, and purification, to achieve the claimed therapeutic effect because of the scarcity of DCs in peripheral circulation. However, the patient refused to undergo traditional DC immunotherapy because he considered it to be time consuming and costly. Therefore, we isolated monocyte-derived dendritic cells (MoDCs) from autologous PB mononuclear cells (PBMCs) without granulocyte colony-stimulating factor mobilization *in vivo* and further expansion *in vitro*, which were later enriched through leukapheresis with PB stem cells (PBSC) collection in the COBE Spectra Apheresis System (7). Next, 4–10 ml of the DC-enriched product was individually and intratumorally injected three times with simultaneous intratumoral injection of durvalumab (anti-PD-L1 antibody). The immunohistochemical stains revealed that PD-L1 expressed in 65% sampled cancer tissue (PD-L1 IHC 28-8 pharmDx Assay, Agilent/Dako). Each dose of 40, 60, and 120 mg of durvalumab was carefully injected into tumor tissue 5 min after each DC-enriched product was injected. Local radiotherapy in the right inguinal region was performed with a total dose of 60 Gy. The total dose was divided into 30 fractions with 2 Gy in each fraction for three treatment courses, which were implemented 5 days after the first and second courses of immunotherapy as well as 9 days before the third course. The main reason we chose the intermittent schedule in TRT for the treatment in this case is to prevent early radiation injury of the immune cells in the radiation field. After the TRT treatment, the patient had mild fever and mild swelling of the right thigh with desquamation and erythematous change. But the previously mentioned symptoms and signs can be relieved after the administration of corticosteroids.

The DC subsets in the purified PB samples were characterized through leukapheresis. The blood DC compartment was identified as Lin^−^HLA-DR^+^ and increased approximately fourfold compared with the PBMC sample without leukapheresis (**Figure 1B**). Moreover, most DC subsets in the DC-enriched PB were CD11c^+^ mDCs (approximately 80%) instead of plasmacytoid DCs (pDCs; approximately 1%) (**Figures 1B, C**, representative data; **Figure 1D**, pooled data for the three replicates). In these DC-enriched PB samples, CD16^+^ mDCs were the major subset in both the overall DC population (**Figure 1C**) and mDC subpopulation, with approximately 80% of the subpopulation being CD16^+^ and only approximately 15% and less than 1% being CD1c^+^ and CD141^+^, respectively (**Figure 1B**, representative data; **Figure 1E**, pooled data for the three replicates). The amounts of injected CD16^+^-predominant mDCs were approximately 7.3 × 10^4^, 2.5 × 10^6^, and 1.7 × 10^7^. The number of injected DCs is relatively lower than other standard protocols that usually had *ex vivo* culture expansion. However, DCs in the peripheral blood of psoriatic patients had been identified and belong to inflammatory DCs (8). Compared with other DCs, those CD16^+^ DCs of psoriatic patients demonstrated superior antigen presentation capacity and drove a strong T cell response (9, 10). Therefore, even if only a few amounts of DCs were injected, it still exerted an immunological effect. According to the current protocol of leukapheresis, the total amount of DCs that can be obtained from peripheral blood is about 7–2,000 × 10^4^. The number of DCs will fluctuate depending on the condition and treatment response. The leukapheresis machine recirculates about 7,000–9,000 ml of the patient’s whole blood into the interface to separate PB mononuclear cells (PBMCs) from other granulocytes and lymphocytes by elutriation. However, only 60–70 ml of concentrated PBMCs was obtained in the collection bag through the leukapheresis. We also remove the excess plasma by centrifuge. We can maximize the volume of recirculating whole blood to produce maximal PBMCs in collection bag but the amount of existing DCs in PB is limited as well as the injectable volume of DCs into tumor mass. These findings demonstrate the potential for CD16^+^ mDCs to be enriched through a specific leukapheresis procedure on PBMCs collected from patients with PV who have developed advanced cSCC.

**Figure 2:**
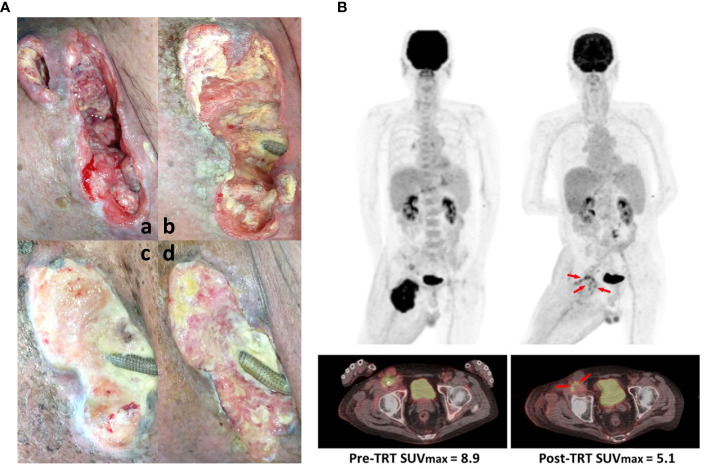
Therapeutic effect of intratumoral immunotherapy combined with local radiation. **(A)** Primary lesion conditions assessed (a) pre-TRT status, (b) after the first TRT course, (c) after the second TRT course, and (d) after the whole TRT period. **(B)** The tumor mass shrank with remarkable SUVmax reduction of 42% on FDG positron emission tomography (PET)-Scan. Tumor sizes before any treatment (left panel) and after the complete TRT period (right panel) were assessed using PET.

To determine the efficacy of the aforementioned immunotherapy plus radiotherapy on our cSCC patient, we first assessed the wound condition using pelvis computed tomography and found that the wound healing process improved gradually with the course of ongoing TRT (**Figure 2A**); the right inguinal mass only measured 5.0 cm × 4.1 cm in the axial dimension and 5 cm in craniocaudal distance compared with pre-TRT imaging. To ascertain the therapeutic effect on the progressive tumor, a PET scan was performed to analyze the tumor size before and after TRT, which indicated a dramatic postintervention change in FDG avidity over the right inguinal mass and right external iliac regions. There was a remarkable SUVmax reduction (approximately 42%) on FDG PET-Scan despite relatively low-dose DCs were available (**Figure 2B**). However, a slight increase in FDG avidity of the paraaortic lymph nodes was noted, suggesting possible lymph node metastasis (LNMets) or immune activation of TRT. These findings support that the combination of immunotherapy—CD16^+^ DC-enriched PB plus anti-PD-L1 antibody—and radiotherapy is effective for treating locally advanced cSCCs with surgical contraindications.

**Figure 3:**
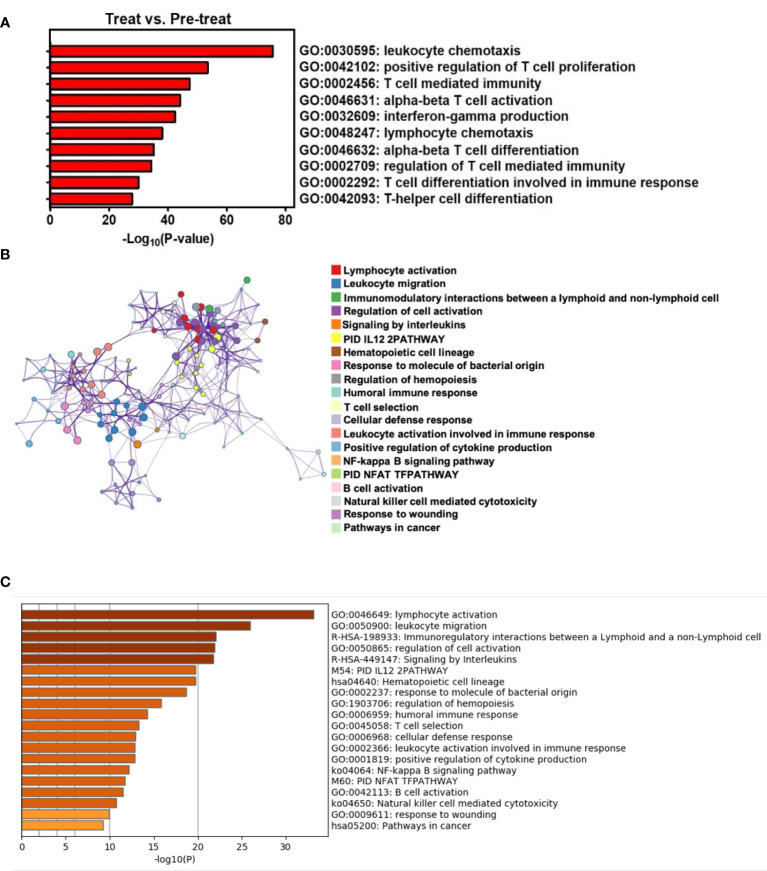
TRT results in enrichment of local T-cell-related pathways and systemic antitumor immunity through Metascape analysis. **(A)** T-cell-related pathways were enriched in the gene ontology biological processes for tumors with TRT versus no treatment by *P*-values. **(B)** The upregulated pathways were composed of differentially expressed genes, presented as a network of enriched terms for peripheral blood with TRT versus no treatment. **(C)** Top 20 clusters of both innate and adaptive immunity-associated pathways were enriched in the gene ontology biology processes for PB with TRT versus no treatment by *P*-values.

To identify the mechanisms underlying this TRT protocol and the potential of DC-mediated immunity, we compared the transcriptomes between pre-TRT and post-TRT biopsies using Metascape (11) to analyze T-cell-related pathways, with over 10 highly significantly enriched pathways (*P <*0.00001) identified in postintervention biopsies. These pathways included the following: leukocyte chemotaxis, positive regulation of T-cell proliferation, T-cell-mediated immunity, alpha-beta T-cell activation, interferon-gamma production, lymphocyte chemotaxis, alpha-beta T-cell differentiation, regulation of T-cell–mediated immunity, T-cell differentiation involved in immune response, and T-helper cell differentiation. These data indicated that T-cell-mediated immunity was locally induced after intratumoral injection with CD16^+^ mDC-enriched cells and anti-PD-L1 therapy (**Figure 3A**). We would like to provide more evidences about the alternations in immune microenvironment of the tumor after TRT, but the tumor samples were insufficient for relevant analysis. However, we analyzed the systemic immune response in pre-TRT and post-TRT PB; the results revealed that the top 20 clusters with corresponding representative terms were enriched in the Gene Ontology Biological Processes Kyoto Encyclopedia of Genes and Genomes Pathways and Reactome gene sets. The network is visualized using Cytoscape5, where the activation of both innate and adaptive immunity was significantly enriched in postintervention PB (**Figure 3B**, colored by cluster pathway; **Figure 3C**, list of top 20 enriched pathways with highly significant *P*-values (*P <*0.00001) for PB with TRT versus no treatment).

These findings suggest that immunotherapy using CD16^+^ mDC-enriched cells plus anti-PD-L1 antibody could be effective for treating advanced cSCC through the upregulation of local T-cell-mediated immune responses and then systemic antitumor immunity that can reduce the tumor size when combined with radiotherapy.

## Discussion

We present a case in which combined radiotherapy and immunotherapy was applied for treating a psoriatic patient with cSCC and contraindications to surgery and chemotherapy, including vascular encasement by the tumor and risk of HBV reactivation.

In addition to contraindication of surgery and chemotherapy, there are remaining several dilemmas in treating the case. First of all, co-existence of cancer and autoimmune disease often created challenging circumstances for doctors and patients. Our patient diagnosed with either disease, these two opposing forces may collide, further complicating treatments and side-effect management efforts. However, substantial evidence implicates that psoriatic patients have an increased risk of squamous cell carcinoma (SCC) (SIR = 5.3, 95% CI 2.63–10.71) (12). Second, cutaneous toxicity is the most prevalent adverse effect of ICIs, and commonly presents as a maculopapular rash, lichenoid skin reactions, *de novo* autoimmune skin disease, or reactivation of previous autoimmune skin diseases, such as psoriasis (13–15). Although several types of immunotherapy are used to treat cancer, patients with autoimmune disease, may have a difficulty tolerating these immunotherapy drugs. Third, there was short of time for decision making because of repeated hypovolemic shock in this case. With the emergent event of pseudoaneurysm over right femoral region complicating aneurysm rupture and twice life-threatening hypovolemic shock, we had no choice but initiate the immediate intervention, instead of time-consuming therapeutic protocol (traditional DC-based tumor vaccine). Finally, the practice guideline of combinational intratumoral injection therapy with low dose autologous MoDCs for management of cSCC is absent.

The best solution to the aforementioned dilemmas is *in situ* vaccination by low-dose DCs and anti-PD-L1 with potentiation by local RT, the combinational strategy obviated the need for high dose boosting of immunotherapy and remitted the dose dependent adverse effects. We therefore checked specific expression levels of PD-L1 expressed in tumor specimen before versus after TRT in our transcriptomic array, and surprisingly found that the expression level of PD-L1 in tumor specimen after TRT was 16.72-fold higher than that before therapy, suggesting that combination of radiotherapy improved the therapeutic efficacy of anti-PD-L1 and DC-based immunotherapy in the cSCC patient. The synergistic effects of combining different immunotherapies, or immunotherapies and RT have been discussed, and showed promise (16, 17). However, to the best of our knowledge no case or preliminary result has thus far addressed the combination therapy of the three regimens.

Describing the role of DCs in the pathogenesis of psoriasis, IL-23/IL-17 axis is vital for Th17-mediated pathogenesis of psoriasis which represents chronic inflammation in skin composed of several types of DCs, namely, plasmacytoid DCs (pDCs), conventional DCs (cDCs), inflammatory DCs, and Langerhans cells (LCs). With external stimuli to cause keratinocyte damage, pDCs are activated *via* self-nucleotide-mediated TLR7 and TLR9 signaling, and then release a huge amount of type I IFNs which can further promote differentiation of peripheral monocytes into CD103^+^ cDC1, CD1c^+^ cDC2 or iDCs. cDC2 and iDCs which include 6-sulfo LacNAc DCs (slan-DCs) and TNF-α and iNOS-producing DCs (Tip-DCs) can secrete IL-23 for enhanced development and expansion of Th17 cells in response to these damage derivatives, while cDC1 can produce IL-2 for Th1 differentiation which may enhance the inflammatory damage. Moreover, IL-1β is produced from damaged keratinocytes to activate LC for IL-23 release and also facilitate Th17 differentiation. The population of slan-DCs have been demonstrated to express CD16 which is considered as an inflammatory DC subset in various types of autoimmune diseases. All of these DC subpopulations then contribute to strong inflammatory T-cell responses and drive psoriasis (8, 9).

CD16^+^ myeloid DCs, which are considered as a critical inflammatory DC subset to secrete cytokines for T-cell activation in autoimmune diseases, are specifically enriched in the cSCC patients who were previously diagnosed with psoriasis. We therefore chose the activated DC subset as the target of immunotherapy to potently improve the T cell immunity against cSCC. Although the role of CD16^+^ myeloid DCs in cSCC pathogenesis is poorly understood, one *in vitro* study demonstrated SCC cell lines can significantly upregulate CD16 expression on DCs with an immature phenotype (18), indicating that SCC may interfere with the function of CD16^+^ DCs but need more *in vitro* and *in vivo* data to explore its role for cSCC pathogenesis in detail. Since T-helper cells have been identified as critical to inhibit SCC growth *in vivo* (19), the functionally reversal cSCC-enriched CD16^+^ DCs by autoimmune psoriasis are likely to facilitate T-cell activation and differentiation into effector T-helper cells as we showed in the present case report. Although there is limited information of CD16^+^ DCs on cSCC, we give an insight into a therapeutic role of functional CD16^+^ DCs for cSCC and provide a convenient protocol for treatment of advanced cSCC.

PD-L1 expression by DCs has been reported as critical in reducing T cell function in cancer (20, 21), which plays a part for cancer development and progression. The combination of anti-PD-L1 and DC-based immunotherapy is therefore becoming a promising immunotherapy (22), while radiotherapy has been confirmed to enhance the therapeutic efficacy of i.t. DCs by induction of IFN-γ-mediated T cell immunity (23). In this report, we aim to concurrently use anti-PD-L1, radiotherapy and DC-based therapy which were simply prepared through leukapheresis for the treatment of the cSCC patient previously diagnosed with psoriasis that may have strong activity of peripheral DCs to increase T cell function (8). At 24 h in peripheral blood with TRT, the frequency of CD4+ T cells was increased with the downregulation of both naïve and activated regulatory T cells (data not shown), indicating the immediate immune response towards inflammatory milieu after TRT. Our data demonstrate multiple T cell-mediated pathways involving activation, proliferation, and IFN-γ production were upregulated with TRT locally as well as the overall immunity enhanced systemically by bioinformatics analyses. Our findings implicated the feasibility of using the TRT for boosting T cell-mediated immunity against carcinoma *in situ*, while enhancing systemic immune response may help to prevent distant metastasis.

We first characterized PBSC apheresis samples derived from the PB of the patient, who had abnormal immune responses due to PV and high levels of CD16^+^ mDCs (24). Because the activation of peripheral CD16^+^ mDCs has been reported to be upregulated by the secretion of inflammatory cytokines in patients with other autoimmune diseases, such as systemic lupus erythematosus (3, 25) and Sjogren syndrome (26), we assessed whether CD16^+^ mDC-enriched cells isolated from the patient were potent for eliciting T cell response through intratumoral injection. *In situ* DC-based immunotherapy was supplemented with anti-PD-L1 and radiation because both measures have been demonstrated to disrupt the growth of tumor bulk and improve the efficacy of immunotherapy through the enhancement of immune responses. In addition, our protocol could produce more tumor antigen-specific DCs than could traditional DC immunotherapy because the mutated tumor antigens are unrecognizable during long-term DC expansion *in vitro*.

It is not excluded that the radiotherapy 1 week after intra-tumor injection of DC-enriched PB leukocytes could kill the injected leukocytes. However, we think that the combination of DCs and intramural-tumor injection of anti-PDL1 might have amplified antigen presentation and built the environment for recruiting systemic T cells one week after injection. Even the regional radiotherapy could kill some of the restimulating T cells, the environment could recruit systemic DCs and T cells to the lesion site. Together with 2nd and 3rd doses of intra-tumor Anti-PDL1 and DC-enriched PB leukocytes, the environment would enforce the mature and immature DCs to take up tumor antigens released after radiotherapy. This is the design to make sure that the combination of intra-tumor DCs injection and regional radiotherapy is effective.

Overall, the therapeutic effect of this TRT on the *in situ* cSCC was acceptable, despite the patient expiring 4 months later due to LNMets. We shared this easily accessible combinational regimen with time-saving, low cost, and less side effect of immunotherapy for treating a psoriatic patient with SCC, refractory to other treatments. Although the cause of the patient’s final death was respiratory failure, which was related to pulmonary edema and acute renal failure, and not related to the deterioration of PV. Except for the tumor shrinkage, the TRT also brought the time saving benefit, unlike the time-consuming therapeutic protocol for traditional DC-based tumor vaccine. It was very important to consider immediate and effective intervention for the patient as he had twice experienced life-threatening hypovolemic shocks.

It is difficult to differentiate the synergistic effects or each individual effects of immunotherapies and/or RT in this case. This huge progressive tumor resistant to chemotherapy would require a better combined therapy rather than radiotherapy alone. Thus, we chose to take advantages of intra-tumor immunotherapy with DC-enriched PB leukocytes and low dosage anti-PDL1 to augment the presentation of the tumor antigens released after radiotherapy. This is a pilot study to take advantages and limit disadvantages of combined regional therapies. It appears to prove the concept that a combination of radiotherapy and intra-tumor immunotherapy with DC-enriched peripheral leukocytes and low dosage anti-PDL1 could limit tumor progression resistant to chemotherapy, presumably through amplifying the antigen presentation of the tumor antigens released after radiotherapy.

In conclusion, we developed an effective DC-based immunotherapy approach based on the enrichment of autologous CD16^+^ mDCs. This immunotherapy protocol was implemented with an anti-PD-L1 plus radiotherapy, which improved the T-cell-mediated immunity and reduced the size of the cSCC. Further studies are warranted to optimize the protocol by increasing the number of injected CD16^+^ mDC-enriched cells.

## Patient Perspectives

Throughout the process, the patient and his wife were informed of treatment options, risk, and the possibility of relapse. They realized the complexity of his unusual case and the patient provided written informed consent for the publication of his case. They appreciated the multidisciplinary group to initiate the affordable TRT to overcome the dilemmas in treating the cancer. After the patient passed away, his wife was happy to agree to publish this case report in hopes of expanding the medical knowledge in this field.

## Data Availability Statement

The original contributions presented in the study are included in the article. Further inquiries can be directed to the corresponding author.

## Ethics Statement

The study involving human participant was reviewed and approved by Tri-Service General Hospital. The protocol was approved with the number 2-108-05-119 on Jul 11, 2018. The patient provided his written informed consent to participate in this study. Written informed consent was obtained from the patient’s family for the publication of any potentially identifiable images or data included in this article.

## Author Contributions

J-WH and C-LK wrote the manuscript and contributed equally to this work as co-first authors. L-TW and KS-KM performed the experiments, analyzed the data and co-wrote the manuscript. W-YH, F-CL and B-HY contributed to the planning, organization of the therapeutic regimen. KDY devised the project, the main conceptual ideas and provided critical edits. B-HY is the corresponding author for this manuscript. All authors provided critical feedback and approved the final manuscript.

## Funding

The study was funded with support of open access publication fees by Ministry of National Defense-Medical Affairs Bureau (MAB106-036, MAB107-023), Teh-Tzer Study Group for Human Medical Research Foundation of Taiwan (B1081050).

## Conflict of Interest

The authors declare that the research was conducted in the absence of any commercial or financial relationships that could be construed as a potential conflict of interest.

## Publisher’s Note

All claims expressed in this article are solely those of the authors and do not necessarily represent those of their affiliated organizations, or those of the publisher, the editors and the reviewers. Any product that may be evaluated in this article, or claim that may be made by its manufacturer, is not guaranteed or endorsed by the publisher.

## Abbreviations

anti-PD-L1, anti-programmed cell death protein-ligand 1; MoDCs, monocyte-derived dendritic cells; DCs, dendritic cells; cSCC, cutaneous squamous cell carcinoma; ICIs, immune checkpoint inhibitors; antiPD-1, anti-programmed cell death protein 1; PD-L, PD-ligand; mDCs, myeloid DCs; PB, peripheral blood; HBV, hepatitis B virus; PET, positron emission tomography; FDG, fluorodeoxyglucose; TRT, triple-regimen therapy; PBMCs, PB mononuclear cells; PBSC, PB stem cells; LNMets, lymph node metastasis.
